# Competing risk nomogram for predicting cancer-specific survival in patients with primary bone diffuse large B-cell lymphoma: a SEER-based retrospective study

**DOI:** 10.3389/fmed.2025.1572919

**Published:** 2025-05-12

**Authors:** Rongbin Shen, Sichun Xiang, Jianyou Gu, Yu Zhang, Lili Qian, Jianping Shen, Qing Guo, Shana Chen, Chenyang Ma, Jingjing Xiang

**Affiliations:** ^1^The First Affiliated Hospital of Zhejiang Chinese Medical University (Zhejiang Provincial Hospital of Traditional Chinese Medicine), Hangzhou, China; ^2^International Mongolian Hospital of Inner Mongolia, Hohhot, China; ^3^Department of Traditional Chinese Medicine, The Second People's Hospital of Xiaoshan District, Hangzhou, China

**Keywords:** primary bone diffuse large B-cell lymphoma, competing risk model, cancer-specific survival, cardiovascular death, nomogram

## Abstract

**Background:**

Cardiovascular death (CVD) represents a significant determinant affecting the long-term survival outcomes of cancer patients, independent of primary tumor effects. Consequently, this study aims to identify prognostic factors in patients with primary bone diffuse large B-cell lymphoma (PB-DLBCL) using CVD as a competing risk and to develop a competing risk nomogram.

**Methods:**

Data for patients diagnosed with PB-DLBCL from 2000 to 2015 were sourced from the Surveillance Epidemiology, and End Results (SEER) database and a total of 1,224 PB-DLBCL patients were eventually included in this study. The approach of multiple imputation is utilized to address the issue of missing data. Univariate Cox regression analysis and the best subset selection method are utilized for variable screening, from which overlapping independent risk factors are identified for subsequent multivariate Cox analysis and multivariate competing risk analysis. The Fine-Gray test was applied for univariate competing risk analysis. Significant variables from the multivariate competing risk analysis were selected as independent prognostic factors to construct a competing risk nomogram for predicting 1-, 5-, and 10-year cancer-specific survival (CSS). The model's performance was evaluated by Harrell concordance index (C-index), time-dependent receiver operating characteristic (ROC) curves, and calibration curves.

**Results:**

Compared with the competing risk model, the conventional Cox regression model overestimates the impact of variables on the incidence of cancer-specific death (CSD). Age, income, B symptoms, Ann Arbor stage, primary site, laterality, chemotherapy, and systemic therapy were identified as independent risk factors for CSD. A competing risk nomogram was developed incorporating these variables to predict CSS. In the training set, the areas under the curve (AUC) for 1-, 5-, and 10-year CSS were 0.879, 0.848, and 0.839, respectively, while in the testing set, the AUC values were 0.794, 0.781, and 0.790, respectively. The C-index of the model was 0.853, 0.823, and 0.819 for 1-, 5-, and 10-year survival in the training set, and 0.777, 0.757, and 0.754 in the testing set. The calibration curve indicated favorable consistency for the competing risk nomogram.

**Conclusions:**

The competing risk nomogram was effectively utilized to predict CSS in patients with PB-DLBCL It exhibited robust predictive performance and holds potential for enhancing treatment decision-making in clinical practice.

## Introduction

Primary bone lymphoma (PBL) is an uncommon malignant lymphoma, initially characterized by Parker and Jackson in 1939 as a distinct clinicopathological entity (1). It accounts for ~1–2% of all lymphomas and 5–7% of all primary malignant bone tumors, with a predilection for the pelvis, spine, and ribs (2–4). The predominant pathological subtype of PBL is non-Hodgkin lymphoma, mainly diffuse large B-cell lymphoma (DLBCL), which accounts for over 80% of cases (5, 6). Primary bone diffuse large B-cell lymphoma (PB-DLBCL) is predominantly of germinal center B-cell (GCB) origin, exhibiting distinct clinical and morphological characteristics, and demonstrates sensitivity to radiotherapy and chemotherapy regimens based on R-CHOP (4, 7–9). Patients with PB-DLBCL generally have a favorable prognosis, with a 5-year overall survival (OS) rate ranging from 60% to 95% (10–14).

Despite recent efforts to comprehensively summarize the clinical characteristics of patients with PB-DLBCL, establish staging systems and prognostic factors, and develop clinical management strategies (15), the rarity of this condition results in most data comes from single-center retrospective studies with limited sample sizes. This limitation, coupled with variability in research outcomes across different centers, renders the factors influencing prognosis still unclear (10, 16). Previous investigations into the prognostic factors of PB-DLBCL have predominantly utilized traditional analytical techniques, such as Cox regression analysis and the Kaplan-Meier method (17, 18). These conventional methods for survival data analysis often fail to account for competing risks—events that preclude the occurrence of the event of interest—potentially leading to an overestimation of the cumulative incidence of the event of interest (19, 20). Therefore, accurately distinguishing causes of death in tumor patients during survival prognosis analysis can improve predictive accuracy and facilitate more informed clinical decision-making.

Due to the combined effects of population growth and aging, along with advancements in early detection and treatment, the survival rates for cancer have improved, resulting in a gradual increase in the number of cancer survivors in the United States (21). Concurrently, the prevalence of non-cancer-related mortality among cancer patients has become increasingly pronounced, particularly given that the risk of cardiovascular diseases is elevated in cancer survivors in comparison to non-cancer patients, thus establishing cardiovascular diseases as a leading cause of mortality within this population, apart from cancer itself (22–24). Considering the generally favorable prognosis and long survival period of patients with PB-DLBCL, performing a competing risk analysis with CVD as a competing risk factor will yield more accurate results.

The Fine-Gray model, introduced by Fine and Gray in 1999, is a statistical approach designed to evaluate the influence of risk factors on individual event types in the context of competing risk events (25). This model provides subdistribution hazard ratios that account for cumulative incidence, offering a more precise risk assessment of the primary outcome compared to traditional survival analysis by addressing complexities from competing events (26, 27).

The Surveillance, Epidemiology, and End Results (SEER) program is a comprehensive cancer statistics database established by the National Cancer Institute, covering ~47.9% of the U.S. population through data collected from diverse geographic areas and populations. It gathers high-quality information on various cancers, including incidence, treatment, and survival rates, and is widely utilized for cancer research and epidemiological studies (28). Using the SEER database, this study applied the Fine-Gray model, incorporating CVD as a competing risk and cancer-specific death (CSD) as the primary event of interest, to evaluate prognostic factors in PB-DLBCL patients and develop a competing risk nomogram for predicting 1-, 5-, and 10-year CSS.

## Materials and methods

### Study population

In this retrospective cohort study, we employed SEER^*^stat software to extract patient data diagnosed with PB-DLBCL from the SEER database (version 8.4.3). Following the third edition of the International Classification of Diseases for Oncology (ICDO-3), we collected data for cases diagnosed between 2000 and 2015 characterized by a histological code of 9680 and primary bone site codes (C40.0–C41.9). The exclusion criteria were as follows: (1) patients younger than 18 years; (2) PB-DLBCL not being the primary malignant tumor; (3) cases identified solely through death certificates or autopsies; (4) unclear disease staging; and (5) outcome status not classified as Alive, CSD, or CVD. Given that the SEER database offers de-identified and publicly accessible data, using these data for research purposes does not require obtaining patient informed consent or ethics review board approval (29).

### Definition of variables

The study examined patient clinical characteristics such as age, race, sex, diagnosis year, marital status, income, Ann Arbor stage, primary site, laterality, B symptoms, radiotherapy, chemotherapy, systemic therapy, presence of other tumors, survival time, and outcome. Income levels were defined as low (<$55,000), medium ($55,000–$74,999), and high (>$75,000). Marital status was categorized into married (including common law), single (never married), and other (separated/divorced/widowed/unmarried or domestic partner/unknown). Systemic therapy was classified as Yes (including post-surgery, both pre- and post-surgery, or unknown sequence) and No (no systemic therapy and/or surgery). The competing risk event was CVD, encompassing aortic aneurysm and dissection, atherosclerosis, cerebrovascular diseases, heart diseases, hypertension without heart disease, and other arterial diseases as recorded in the SEER database (30).

### Missing data

Multiple Imputation (MI) is a common method for handling missing values via repeated simulations. It creates multiple complete datasets from an original dataset with missing values, using the Monte Carlo method for imputation. MI aims to generate reasonable estimates that reflect uncertainty while preserving key data relationships and distributions (31). In this study, the variables with missing data include Age (*n* = 1), Race (*n* = 7), Radiation (*n* = 33), Marital status (*n* = 54), Systemic therapy (*n* = 419), and B Symptoms (*n* = 739), totaling 1253 cases, which accounts for 6% of the total data (Supplementary Figure S1). The methods “pmm,” “polyreg,” “logreg,” “polyreg,” “logreg,” and “logreg” were applied to the variables Age, Race, Radiation, Marital status, Systemic therapy, and B Symptoms, respectively. A total of six imputations were performed. Finally, the most suitable dataset for subsequent analysis was selected using the Akaike Information Criterion (AIC) criterion (32).

### Statistical analysis

The X-tile software was utilized to determine the optimal cutoff point for the continuous variable (age), converting it into an ordinal categorical variable (33). The X-tile algorithm systematically evaluates each potential cutoff value across the variable's range. For each candidate value, it calculates corresponding χ^2^ statistics (or log-rank statistics for survival data) and *P*-values based on contingency table analysis. The optimal cutoff is determined by identifying the value that maximizes χ^2^ while minimizing the *P*-value, ensuring statistically robust stratification of the variable. The data were randomly divided into a training set and a testing set at a ratio of 7:3. Categorical variables were presented as frequencies and percentages, and statistical comparisons between groups were conducted using the Chi-square test or Fisher's exact test.

First, for competing risk data, the optimal subset selection method will be employed to identify variables in the training set. Subsequently, the CVD outcomes in the training set will be converted to censored, transforming the data into standard survival data for variable selection using univariate Cox regression. The common variables identified from both the univariate Cox regression analysis with statistical significance (*P* < 0.05) and the optimal subset selection method will be subjected to multivariate Cox regression analysis and multivariate competing risk analysis, respectively. Univariate analysis of competing risk data was conducted using the cumulative incidence function (CIF), and differences between groups were assessed with Gray's test. The Fine-Gray model was used to perform multivariate analysis on the variables selected by the optimal subset method, with the Subdistribution Hazard Ratio (SHR) employed to describe the effect of covariates on the risk of CSD. The identified significant variables were incorporated into the construction of a competing risk nomogram to predict 1-, 5-, and 10-year survival probabilities. Finally, the nomogram was validated using the testing set, and its predictive performance in both the training and testing sets was evaluated through calibration curves, the C-index, and ROC curves.

All statistical analyses were conducted using R software (version 4.3.1; http://www.r-project.org/). Statistical significance was defined as a two-sided *P*-value <0.05. The “mice” (34) package was used for multiple imputation of missing data, the “survival” (35) package for Cox regression analysis, and the “forestplot” (36) package for generating forest plots. The “cmprsk,” (37) “riskRegression,” (38) and “prodlim” (39) packages were utilized for competing risk analysis, while the “mstate” (40) and “rms” (41) packages were employed for constructing the competing risk nomogram. The “pec” (42) package was used for generating calibration curves and calculating the concordance index (C-index). The“ggplot2” (43) package was utilized for data visualization.

## Results

### Population baseline characteristics

**Figure 1:**
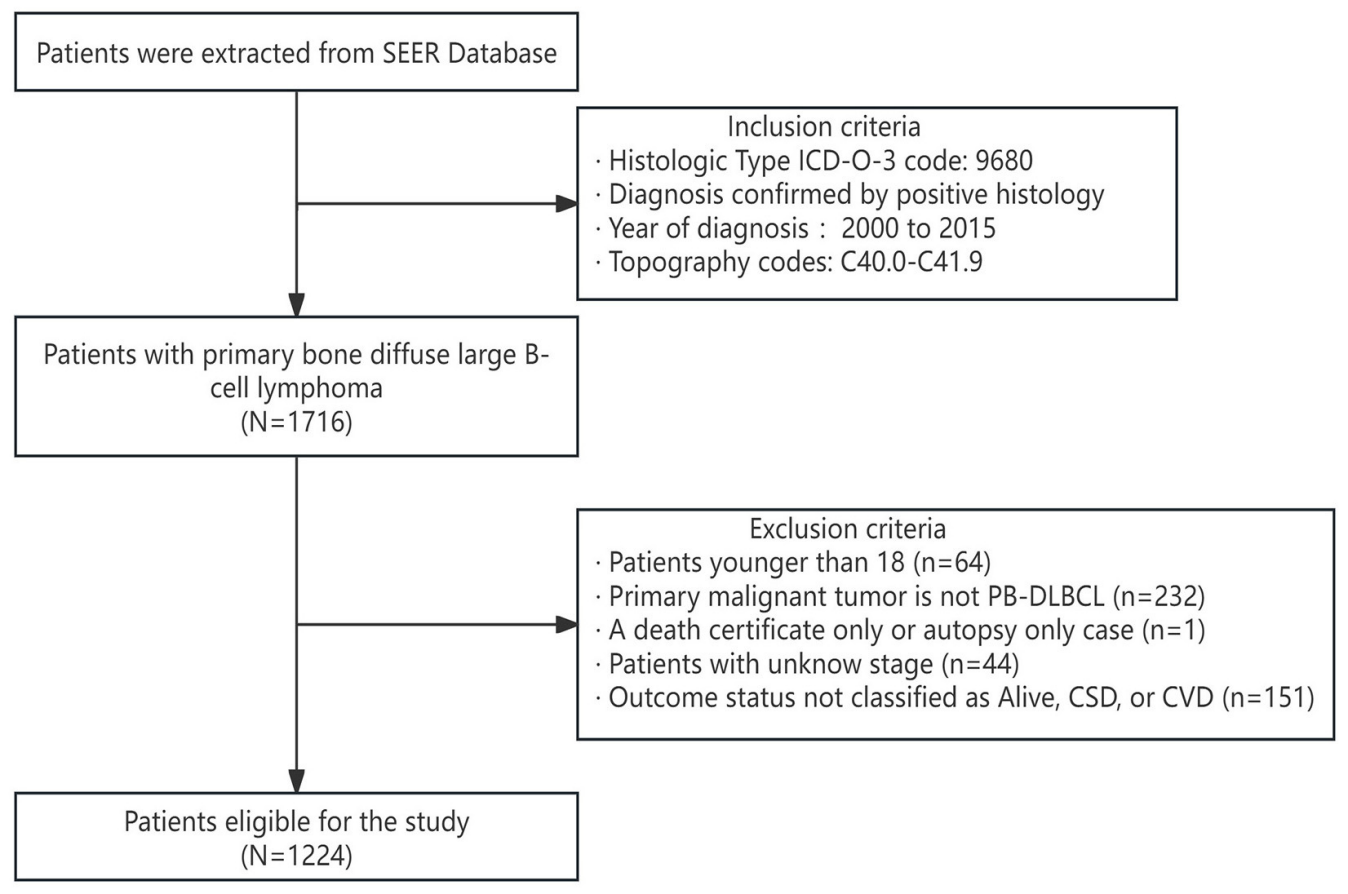
Patient selection flowchart.

**Figure 2:**
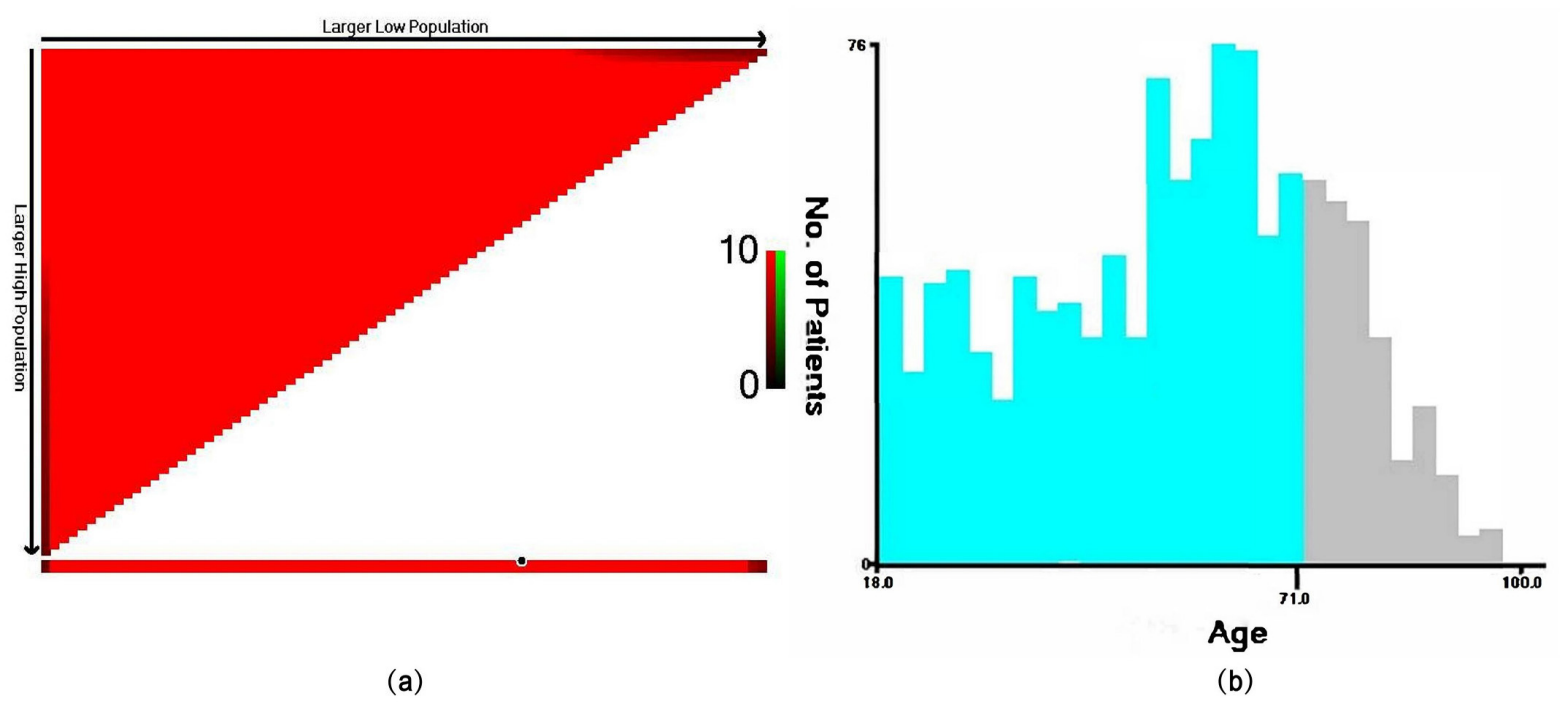
The optimal cutoff value for age determined by the X-tile software. In the left plot **(a)**, the *x*-axis of the triangle represents all potential cutoff points from low to high (from left to right), while the *y*-axis represents the cutoff points from high to low (from top to bottom). Red coloration of cut-points indicates an inverse correlation with survival, whereas green coloration represents direct associations. Analysis of the survival data with X-tile reveals optimal cut-points of (18–71 and ≥72 years), as shown in plot **(b)**.

We extracted a total of 1,224 eligible patients from the SEER database, and the detailed flowchart is shown in Figure 1. Based on the analysis conducted using X-tile, the optimal cutoff value for age was determined to be 71 years, dividing the patients into two groups: 18–71 years and ≥72 years, as shown in Figure 2. The baseline characteristics of all patients are shown in Table 1, with 859 patients comprising the training set and 365 patients comprising the testing set. Among all patients, there were 655 males (53.5%) and 569 females (46.5%). The majority were white, accounting for 87.2%. Married individuals comprised the majority at 56.7%. The group with medium income accounted for 48.7%. Those with B symptoms accounted for 67.9%. In terms of Ann Arbor stage, the largest group was in stage I, representing 54.4%, followed by stage IV at 31.6%. In the primary sites of the tumor, of the bones involved, the limb was the most affected, accounting for 38.2%, followed by the vertebral region at 27.6%. 86% of the patients received chemotherapy. 57.6% of the patients received radiation therapy. Only 20.8% of the patients received systemic therapy. The vast majority of patients have only one primary tumor, PB-DLBCL, accounting for 90.8%.The median follow-up period was 89 (34–147) months, with a maximum duration of 239 months.

**Table 1:** Baseline characteristics of patients with PB-DLBCL.

**Characteristics**	**Overall (*N =* 1,224)**	**Training set (*N =* 859)**	**Testing set (*N =* 365)**	***P*-value**
**Year of diagnosis**				
2000–2003	235 (19.2%)	157 (18.3%)	78 (21.4%)	
2004–2007	286 (23.4%)	198 (23.1%)	88 (24.1%)	
2008–2011	345 (28.2%)	239 (27.8%)	106 (29.0%)	
2012–2015	358 (29.2%)	265 (30.8%)	93 (25.5%)	0.255
**Age**				
18–71	918 (75.0%)	635 (73.9%)	283 (77.5%)	
≥72	306 (25.0%)	224 (26.1%)	82 (22.5%)	0.182
**Sex**				
Female	569 (46.5%)	410 (47.7%)	159 (43.6%)	
Male	655 (53.5%)	449 (52.3%)	206 (56.4%)	0.181
**Race**				
Other	69 (5.6%)	44 (5.1%)	25 (6.8%)	
White	1067 (87.2%)	755 (87.9%)	312 (85.5%)	
Black	88 (7.2%)	60 (7.0%)	28 (7.7%)	0.429
**Marital status**				
Single	287 (23.4%)	210 (24.4%)	77 (21.1%)	
Married	694 (56.7%)	480 (55.9%)	214 (58.6%)	
Other	243 (19.9%)	169 (19.7%)	74 (20.3%)	0.446
**Income**				
Low income	241 (19.7%)	165 (19.2%)	76 (20.8%)	
Medium income	596 (48.7%)	430 (50.1%)	166 (45.5%)	
High income	387 (31.6%)	264 (30.7%)	123 (33.7%)	0.341
**B Symptoms**				
No	831 (67.9%)	594 (69.2%)	237 (64.9%)	
Yes	393 (32.1%)	265 (30.8%)	128 (35.1%)	0.148
**Ann Arbor Stage**				
Stage I	666 (54.4%)	465 (54.1%)	201 (55.1%)	
Stage II	146 (11.9%)	107 (12.5%)	39 (10.7%)	
Stage III	25 (2.0%)	21 (2.4%)	4 (1.1%)	
Stage IV	387 (31.6%)	266 (31.0%)	121 (33.2%)	0.336
**Primary site**				
Cranium	108 (8.8%)	77 (9.0%)	31 (8.5%)	
Limb	467 (38.2%)	335 (39.0%)	132 (36.2%)	
NOS	98 (8.0%)	73 (8.5%)	25 (6.8%)	
Overlap	7 (0.6%)	5 (0.6%)	2 (0.5%)	
Pelvic	161 (13.2%)	113 (13.2%)	48 (13.2%)	
Rib	45 (3.7%)	27 (3.1%)	18 (4.9%)	
Vertebral	338 (27.6%)	229 (26.7%)	109 (29.9%)	0.592
**Laterality**				
Other	10 (0.8%)	6 (0.7%)	4 (1.1%)	
No paired	630 (51.5%)	434 (50.5%)	196 (53.7%)	
Left	306 (25.0%)	214 (24.9%)	92 (25.2%)	
Right	278 (22.7%)	205 (23.9%)	73 (20.0%)	0.439
**Chemotherapy**				
No	171 (14.0%)	114 (13.3%)	57 (15.6%)	
Yes	1053 (86.0%)	745 (86.7%)	308 (84.4%)	0.279
**Systemic therapy**				
No	969 (79.2%)	686 (79.9%)	283 (77.5%)	
Yes	255 (20.8%)	173 (20.1%)	82 (22.5%)	0.359
**Radiation**				
No	519 (42.4%)	369 (43.0%)	150 (41.1%)	
Yes	705 (57.6%)	490 (57.0%)	215 (58.9%)	0.547
**Other tumors**				
No	1112 (90.8%)	781 (90.9%)	331 (90.7%)	
Yes	112 (9.2%)	78 (9.1%)	34 (9.3%)	0.896
**Status**				
Alive	823 (67.2%)	577 (67.2%)	246 (67.4%)	
CSD	312 (25.5%)	219 (25.5%)	93 (25.5%)	
CVD	89 (7.3%)	63 (7.3%)	26 (7.1%)	0.991

NOS, not otherwise specified.

During the follow-up period, 312 (25.5%) patients died from PB-DLBCL, while 89 (7.3%) patients died from cardiovascular diseases. The Chi-square test indicated no statistically significant differences (*P* > 0.05) between all variables in the training and testing sets, affirming comparability at baseline.

### Competing risk model vs. traditional cox regression model

The best subset selection method was employed for variable selection in the training set. Based on the AIC, the final model incorporated 10 variables: race, age, income, B symptoms, Ann Arbor stage, primary site, laterality, chemotherapy, systemic therapy, and radiation. Convert CVD to censoring and conduct univariate Cox regression analysis. The results in Table 2 show that the independent prognostic factors related to CSD in the training set are sex, marital status, age, income, B symptoms, Ann Arbor stage, primary site, laterality, chemotherapy, and systemic therapy (*P* < 0.05). The common significant variables between the two methods are shown in Supplementary Figure S2, which include age, Income, B symptoms, Ann Arbor stage, primary site, laterality, chemotherapy, and systemic therapy. Based on the aforementioned variables, we conducted multivariate Cox regression analysis and multivariate competing risk analysis in the training set.

**Table 2:** Univariate Cox regression analysis for CVD of PB-DLBCL in the training set.

**Characteristic**	***N***	**HR (95%CI)**	***P*-value**
**Sex**			
Female	410	Reference	
Male	449	0.686[0.525,0.895]	0.006
**Race**			
Other	44	Reference	
White	755	0.760[0.442,1.308]	0.322
Black	60	0.648[0.309,1.359]	0.251
**Marital status**			
Single	210	Reference	
Married	480	1.562[1.060,2.302]	0.024
Other	169	3.510[2.325,5.298]	<0.001
**Age**			
18-71	635	Reference	
≥72	224	6.238[4.748,8.196]	<0.001
**Income**			
Low income	165	Reference	
Medium income	430	0.687[0.499,0.947]	0.022
High income	264	0.534[0.368,0.775]	0.001
**B symptoms**			
No	594	Reference	
Yes	265	1.777[1.359,2.323]	<0.001
**Ann Arbor stage**			
Stage I	465	Reference	
Stage II	107	0.947[0.598,1.501]	0.816
Stage III	21	1.415[0.621,3.224]	0.408
Stage IV	266	1.644[1.237,2.185]	0.001
**Primary site**			
Cranium	77	Reference	
Limb	335	1.119[0.584,2.145]	0.735
NOS	73	2.037[0.969,4.280]	0.06
Overlap	5	5.633[1.569,20.219]	0.008
Pelvic	113	2.352[1.189,4.654]	0.014
Rib	27	2.579[1.069,6.225]	0.035
Vertebral	229	3.454[1.848,6.456]	<0.001
**Laterality**			
Other	6	Reference	
No paired	434	0.295[0.109,0.797]	0.016
Left	214	0.156[0.056,0.436]	<0.001
Right	205	0.120[0.042,0.340]	<0.001
**Chemotherapy**			
No	114	Reference	
Yes	745	0.246[0.183,0.331]	<0.001
**Systemic therapy**			
No	686	Reference	
Yes	173	0.491[0.330,0.731]	<0.001
**Radiation**			
No	369	Reference	
Yes	490	0.928[0.710, 1.214]	0.587
**Other tumors**			
No	781	Reference	
Yes	78	0.741[0.452, 1.216]	0.235

HR, hazard ratio; CI, confidence interval.

The results showed that in the multivariate Cox regression analysis, the subgroups of variables that were statistically significant were as follows: age ≥ 72 years (HR = 6.104, 95% CI 4.595–8.108, *P* < 0.001); high income (HR = 0.604, 95% CI 0.411–0.887, *P* = 0.001); presence of B symptoms (HR = 1.759, 95% CI 1.328–2.330, *P* < 0.001); stage IV (HR = 1.750, 95% CI 1.281–2.390, *P* < 0.001); overlap (HR = 7.303, 95% CI 1.947–27.402, *P* = 0.003); pelvic (HR = 2.750, 95% CI 1.323–5.715, *P* = 0.007); rib (HR = 3.143, 95% CI 1.265–7.806, *P* = 0.014); vertebral (HR = 3.410, 95% CI 1.797–6.473, *P* < 0.001); right (HR = 0.253, 95% CI 0.085–0.756, *P* = 0.014); receiving chemotherapy (HR = 0.308, 95% CI 0.220–0.430, *P* < 0.001); and receiving systemic therapy (HR = 0.601, 95% CI 0.390–0.928, *P* = 0.022). However, in the multivariate competing risk analysis, the subgroups of variables that were statistically significant were as follows: age ≥ 72 years (SHR = 5.161, 95% CI 3.848–6.922, *P* < 0.001); high income (SHR = 0.537, 95% CI 0.361–0.797, *P* = 0.001); presence of B symptoms (SHR = 1.768, 95% CI 1.311–2.385, *P* = 0.002); stage IV (SHR = 1.589, 95% CI 1.139–2.215, *P* = 0.006); overlap (SHR = 5.955, 95% CI 2.352–15.078, *P* < 0.001); rib (SHR = 2.691, 95% CI 1.065–6.800, *P* = 0.036); vertebral (SHR = 3.185, 95% CI 1.685–6.019, *P* < 0.001); no paired (SHR = 0.401, 95% CI 0.175–0.918, *P* = 0.031); left (SHR = 0.395, 95% CI 0.172–0.903, *P* = 0.028); right (SHR = 0.257, 95% CI 0.110–0.599, *P* = 0.002); receiving chemotherapy (SHR = 0.405, 95% CI 0.280–0.586, *P* < 0.001); and receiving systemic therapy (SHR = 0.627, 95% CI 0.405–0.970, *P* = 0.036) (Table 3).

**Table 3:** Results of the multivariate Cox regression analysis and multivariate competing risk analysis of the training set.

**Characteristic**	***N***	**Multivariate COX regression**		**Fine-gray**	
		**HR (95%CI)**	***P-*** **value**	**SHR (95%CI)**	***P-*** **value**
**Age**					
18-71	635	Reference		Reference	
≥72	224	6.104 [4.595, 8.108]	<0.001	5.161 [3.848, 6.922]	<0.001
**Income**					
Low income	165	Reference		Reference	
Medium income	430	0.723 [0.520, 1.005]	0.053	0.718 [0.508, 1.013]	0.059
High income	264	0.604 [0.411, 0.887]	0.01	0.537 [0.361, 0.797]	0.002
**B symptoms**					
No	594	Reference		Reference	
Yes	265	1.759 [1.328, 2.330]	<0.001	1.768 [1.311, 2.385]	<0.001
**Ann Arbor stage**					
Stage I	465	Reference		Reference	
Stage II	107	1.178 [0.727, 1.910]	0.507	1.124 [0.676, 1.872]	0.652
Stage III	21	0.652 [0.281, 1.516]	0.321	0.764 [0.315, 1.852]	0.552
Stage IV	266	1.750 [1.281, 2.390]	<0.001	1.589 [1.139, 2.215]	0.006
**Primary site**					
Cranium	77	Reference		Reference	
Limb	335	1.916 [0.850, 4.322]	0.117	1.515 [0.703, 3.262]	0.289
NOS	73	1.638 [0.742, 3.619]	0.222	1.635 [0.744, 3.591]	0.221
Overlap	5	7.303 [1.947, 27.402]	0.003	5.955 [2.352, 15.078]	<0.001
Pelvic	113	2.750 [1.323, 5.715]	0.007	2.049 [0.981, 4.280]	0.056
Rib	27	3.143 [1.265, 7.806]	0.014	2.691 [1.065, 6.800]	0.036
Vertebral	229	3.410 [1.797, 6.473]	<0.001	3.185 [1.685, 6.019]	<0.001
**Laterality**					
Other	6	Reference		Reference	
No paired	434	0.506 [0.173, 1.478]	0.213	0.401 [0.175, 0.918]	0.031
Left	214	0.413 [0.141, 1.208]	0.106	0.395 [0.172, 0.903]	0.028
Right	205	0.253 [0.085, 0.756]	0.014	0.257 [0.110, 0.599]	0.002
**Chemotherapy**					
No	114	Reference		Reference	
Yes	745	0.308 [0.220, 0.430]	<0.001	0.405 [0.280, 0.586]	<0.001
**Systemic therapy**					
No	686	Reference		Reference	
Yes	173	0.601 [0.390, 0.928]	0.022	0.627 [0.405, 0.970]	0.036

SHR, Sub-distribution hazards ratio.

### Univariate competing risk analysis

**Figure 3:**
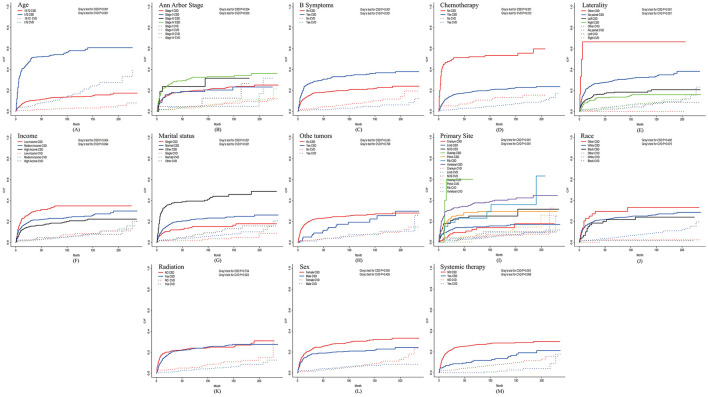
Curves of CIF of CSD and CVD in patients with PB-DLBCL. **(A)** Age; **(B)** Ann Arbor stage; **(C)** B Symptoms; **(D)** Chemotherapy; **(E)** Laterality; **(F)** Income; **(G)** Marital status; **(H)** Othe tumors; **(I)** Primary Site; **(J)** Race; **(K)** Radiation; **(L)** Sex; **(M)** Systemic therapy. The solid line represents CSD, while the dashed line represents CVD. Fine-Gray test is used to statistically assess the differences among groups for all factors and provides a *P*-value.

The univariate analysis of all variables in the training set was performed using the Fine-Gray test, and the CIF curves for CSD and CVD were plotted (Figure 3). As shown in Figure 3, the CIF of CSD exhibits statistically significant differences among the subgroups of these variables: age (*P* < 0.001), sex (*P* = 0.005), marital status (*P* < 0.001), income (*P* = 0.004), B symptoms (*P* < 0.001), Ann Arbor stage (*P* = 0.004), primary site (*P* < 0.001), laterality (*P* < 0.001), chemotherapy (*P* < 0.001), and systemic therapy (*P* < 0.001). Among them, the subgroups of other tumors and the subgroups of race showed no statistical differences in the CIF of both CSD and CVD. Notably, the subgroups of radiation (*P* = 0.025) exhibited statistical differences only in the CIF of the CVD.

### Competing risk nomogram construction and validation

**Figure 4:**
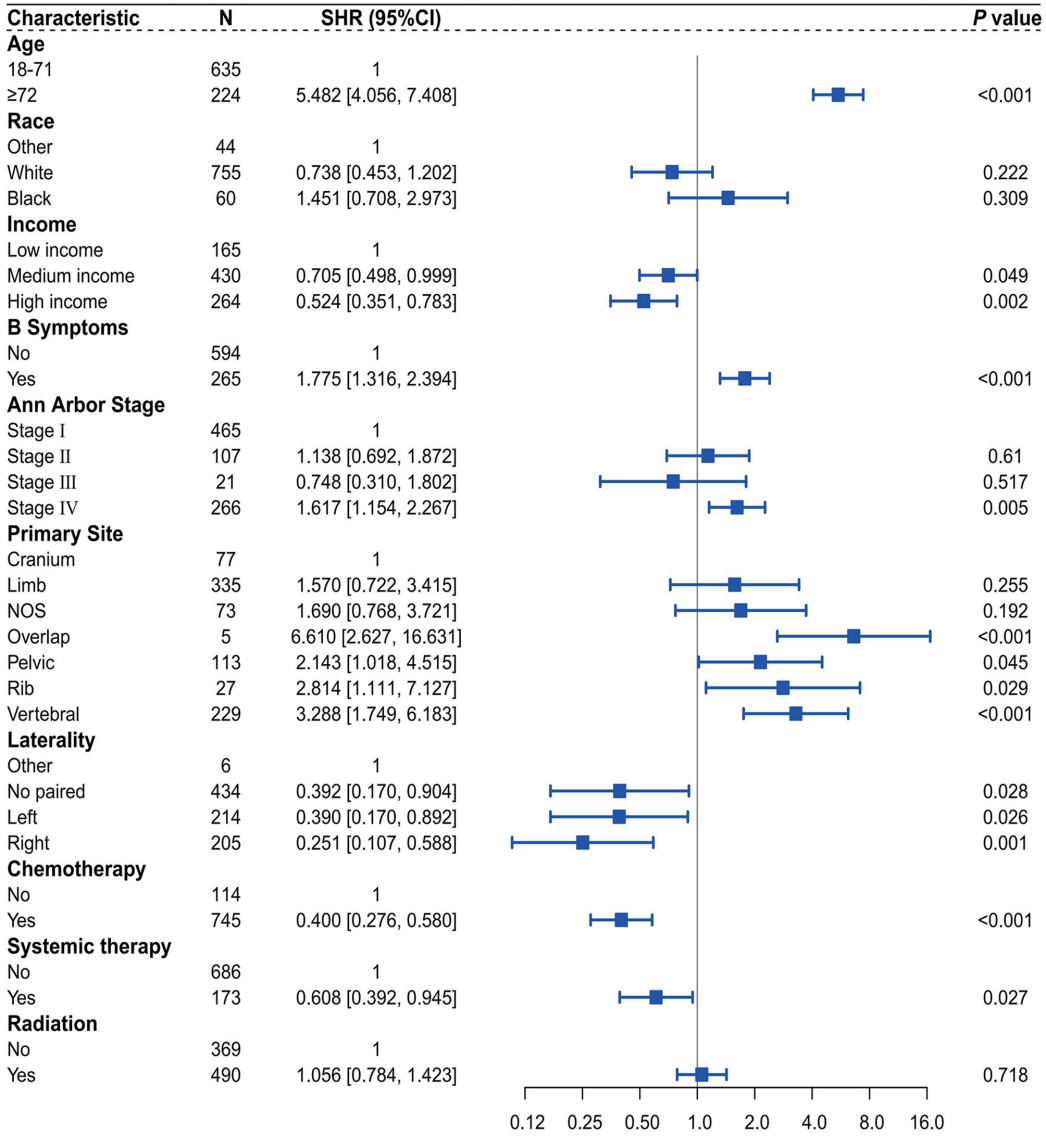
Forest plot of multivariate competing risk analysis of the Fine-Gray Model.

Initially, within the training dataset, a multivariable competing risk analysis was conducted utilizing the Fine-Gray model on 10 variables identified through the optimal subset selection method, which included race, age, income, B symptoms, Ann Arbor stage, primary site, laterality, chemotherapy, systemic therapy, and radiation (Figure 4). The results indicate that, after controlling for the competing risk, patients aged ≥72 years demonstrate statistical significance in their impact on CSD compared to those aged 18–71 years (SHR = 5.482, 95% CI 4.056–7.408, *P* < 0.001). Additionally, patients with high income and medium income show statistical significance in their impact on CSD compared to those with low income, with SHR values of 0.524 (95% CI 0.351–0.783, *P* = 0.002) and 0.705 (95% CI 0.498–0.999, *P* = 0.049), respectively. The presence of B symptoms is statistically significant in comparison to the absence of B symptoms (SHR = 1.775, 95% CI 1.316–2.394, *P* < 0.001). Similarly, patients in stage IV exhibit statistical significance regarding their impact on CSD compared to those in stage I (SHR = 1.617, 95% CI 1.154–2.267, *P* = 0.005). Regarding tumor primary site, patients with tumors located in the overlap, pelvic, rib, and vertebral areas demonstrate statistical significance in their impact on CSD compared to those with tumors in the cranium, with SHR values of 6.610 (95% CI 2.627–16.631, *P* < 0.001), 2.143 (95% CI 1.018–4.515, *P* = 0.045), 2.814 (95% CI 1.111–7.127, *P* = 0.029), and 3.288 (95% CI 1.749–6.183, *P* < 0.001), respectively. In terms of tumor laterality, variables showing statistical significance in their impact on CSD compared to the other subgroup include no paired (SHR = 0.392, 95% CI 0.170–0.904, *P* = 0.028), left (SHR = 2.814, 95% CI 1.111–7.127, *P* = 0.029), and right (SHR = 0.251, 95% CI 0.107–0.588, *P* = 0.001). Patients who received chemotherapy demonstrate statistical significance in their impact on CSD compared to those who did not receive chemotherapy (SHR = 0.400, 95% CI 0.276–0.580, *P* < 0.001). Patients who received systemic therapy show statistical significance in their impact on CSD compared to those who did not receive such treatment (SHR = 0.608, 95% CI 0.392–0.945, *P* = 0.027).

**Figure 5:**
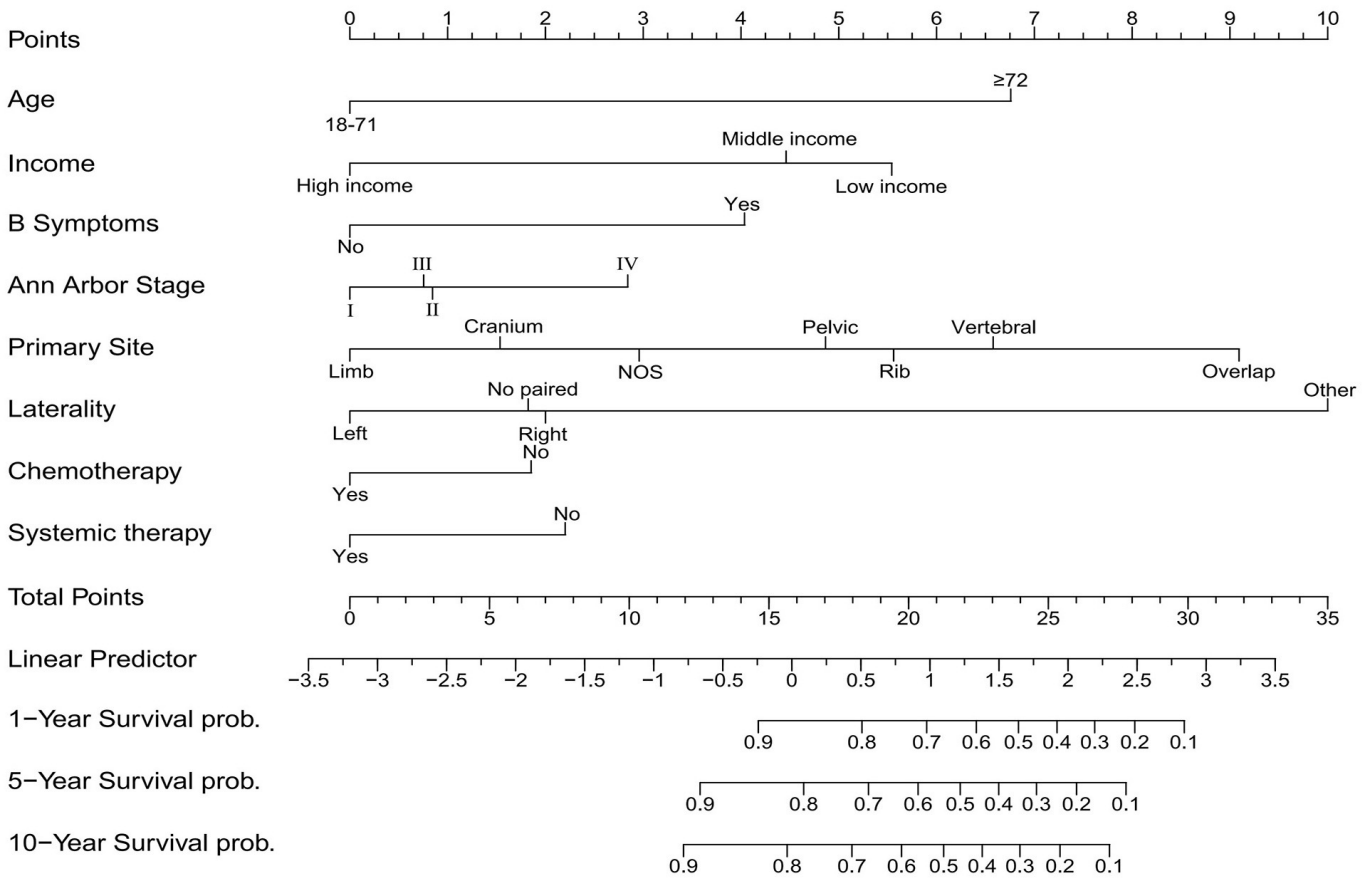
Nomogram based on the competing risk model.

**Figure 6:**
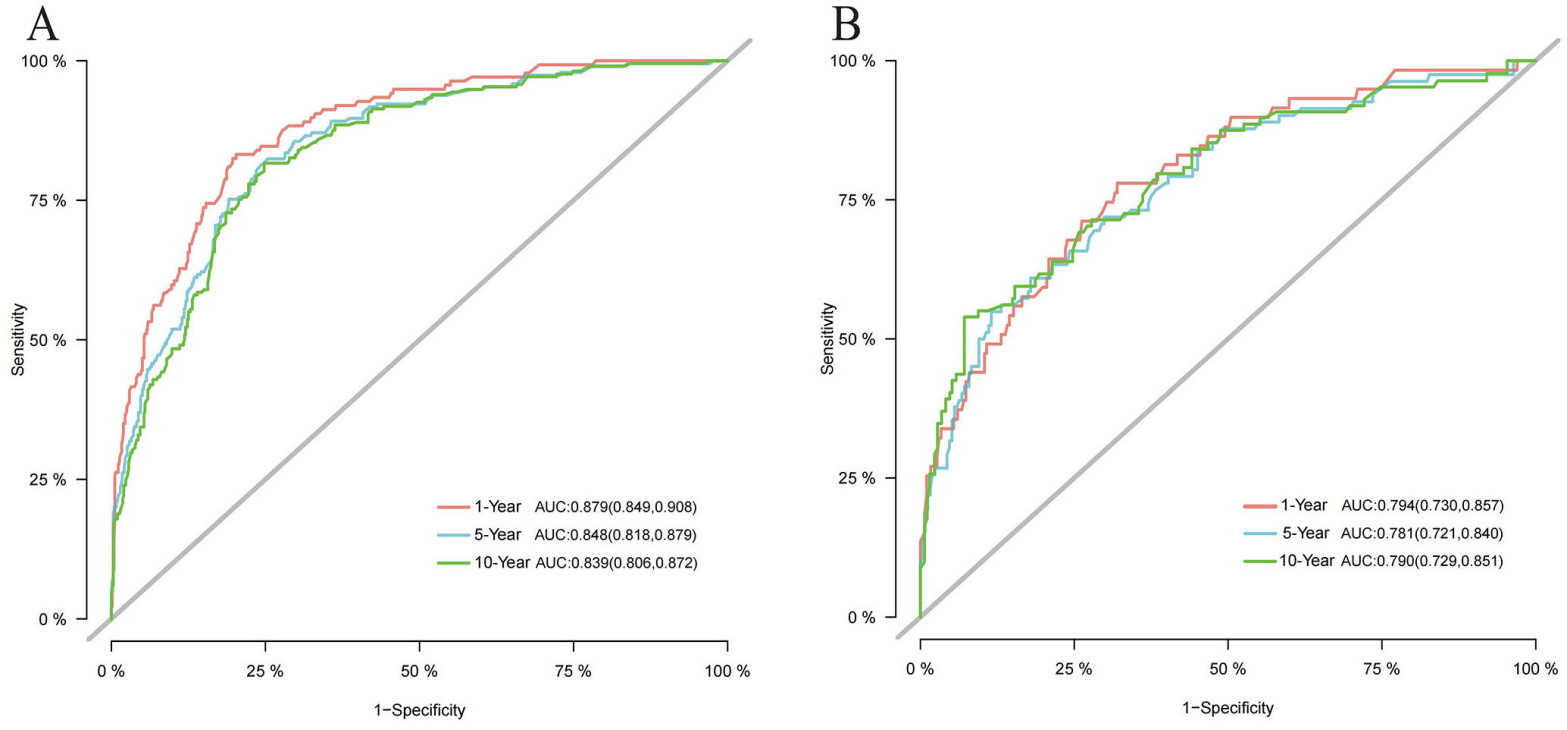
Time-dependent ROC curves of the competing risk nomogram for predicting CSS at 1-, 5-, and 10-year. **(A)** ROC curves at 1-, 5-, and 10 year in the training set; **(B)** ROC curves at 1-, 5-, and 10 year in the testing set.

**Figure 7:**
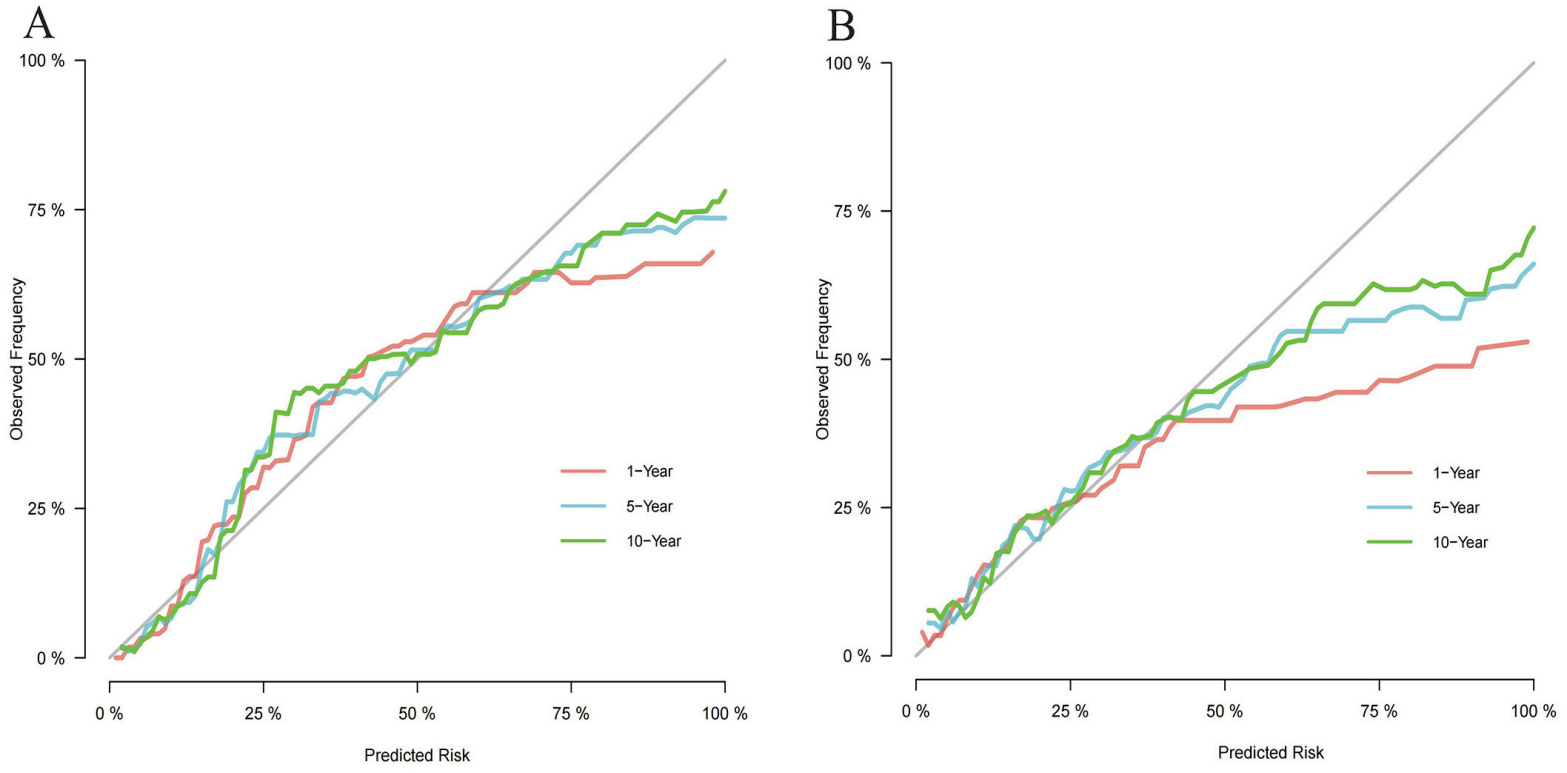
Calibration curves of the competing risk nomogram for predicting CSS at 1-, 5-, and 10-year. **(A)** Calibration curves at 1-, 5-, and 10-year in the training set; **(B)** Calibration curves at 1-, 5-, and 10-year in the testing set.

We utilized independent risk factors identified through multivariate competing risk analysis (including age, income, B symptoms, Ann Arbor stage, primary site, laterality, chemotherapy, and systemic therapy) to construct a nomogram in the training set for predicting 1-, 5-, and 10-year CSS in patients with PB-DLBCL (Figure 5). The C-indices of the competing risk nomogram for predicting 1-, 5-, and 10-year CSS in the training and testing sets were 0.853, 0.823, 0.819 and 0.777, 0.757, 0.754, respectively. In the training set, the ROC curves for predicting 1-, 5-, and 10-year CSS using the competing risk nomogram, illustrated in Figure 6A, exhibited AUCs of 0.879 (95% CI 0.849–0.908), 0.848 (95% CI 0.818–0.879), and 0.839 (95% CI 0.806–0.872), while the calibration curves in the testing set, presented in Figure 6B, demonstrated AUCs of 0.794 (95% CI 0.730–0.857), 0.781 (95% CI 0.721–0.840), and 0.790 (95% CI 0.729–0.851). Figure 7A displays the calibration curves for the competing risk nomogram predicting 1-, 5-, and 10-year outcomes in the training set, while Figure 7B shows the calibration curves for the same predictions in the testing set, with both demonstrating excellent consistency.

## Discussion

We utilized the Fine-Gray model, considering CVD as the competing risk event and CSD as the event of interest, to conduct a competing risk analysis for 1,224 patients diagnosed with PB-DLBCL in the SEER database from 2000 to 2015. A comparative analysis of the results obtained through the multivariate competing risk model and multivariate Cox regression further corroborated the previous conclusions. The results of the multivariate competing risk analysis showed that age ≥72 years, B symptoms, stage IV, and tumors located in the overlap, pelvic, rib, and vertebral areas are independent risk factors for CSD, while no paired, left or right tumor laterality, higher household income, receiving chemotherapy, and receiving systemic therapy are independent protective factors. We successfully constructed and validated a nomogram based on the aforementioned competing risk model, which includes variables such as age, income, B symptoms, Ann Arbor stage, primary site, laterality, chemotherapy, and systemic therapy, to predict CSS at 1, 5, and 10 years. The nomogram achieved an AUC of over 0.7 in both the training set and testing set, indicating good accuracy. The calibration curve and a high C-index also demonstrate the nomogram's reasonable consistency.

To better compare the traditional Cox proportional hazards model with the competing risk model, we used univariable Cox analysis and optimal subset selection methods to screen variables, selecting the overlapping variables for multivariable analysis for comparison. The results revealed that, in the majority of cases, when the HR or SHR of a variable exceeds 1, indicating that the variable functions as a risk factor, the HR value surpasses the SHR. Conversely, when the HR or SHR is <1, signifying that the variable acts as a protective factor, the HR value is inferior to the SHR. In other words, when competing risk events are present, traditional Cox regression models tend to overestimate the impact of covariates on the incidence rate of outcomes compared to competing risk models, which is consistent with previous research results (26).

Our research indicates that age is a significant prognostic factor. In the previous study by Wang et al., compared to patients aged <60 years, the death risk of DSS and OS for PBL patients aged over 75 years and those aged 61–75 years was 2–7 times higher (44), which is consistent with our study. After controlling for the competing risk event, patients aged ≥72 years with PB-DLBCL demonstrated a 4.482-fold increase in CSD risk compared to patients aged 18–71 years. This may be related to the prevalence of multiple comorbidities in older patients, their generally poorer physical condition, and a reduced tolerance to chemotherapy (44, 45).

Medium income and high income are recognized as protective factors. Considering the significant financial burden associated with cancer treatment, patients with higher income are typically able to access superior and more comprehensive treatment and care. Additionally, high income patients often undergo screenings more frequently, allowing for earlier detection of tumors. In contrast, low-income individuals are often diagnosed at more advanced stages of cancer, resulting in poorer treatment outcomes, and may not receive adequate treatment due to financial constraints (46–48).

B symptoms refer to a series of systemic symptoms, including unexplained fever >38°C, night sweats, and weight loss of more than 10% within the past 6 months. The presence of B symptoms is a marker for more advanced disease with systemic, rather than merely local, involvement. The results of this study indicate that after controlling for competing risk events, patients with B symptoms have a risk of CSD that is 1.775 times greater than that of patients without B symptoms. Similarly, in previous studies, B symptoms have been regarded as a significant negative prognostic factor in diffuse large B-cell lymphoma (49, 50).

Additionally, after controlling for the competing risk event, the risk of CSD in stage IV patients is increased by 61.7% compared to stage I patients, consistent with previous studies that consider higher Ann Arbor Stage to be an unfavorable factor for PBL patients (51). In the International Prognostic Index (IPI) for non-Hodgkin lymphoma (NHL), staging at III or IV is recognized as an indicator of poor prognosis. However, this study did not observe a statistically significant correlation between stage III and survival rates (*P* > 0.05). This lack of significance may be attributable to the small sample size of stage III patients in training set, which included only 21 cases (2.4%), thereby limiting the ability to effectively assess the impact of this variable on survival outcomes. Among the primary tumor sites, areas such as overlap, pelvic, rib, and vertebral regions are identified as risk factors compared to the cranium. Notably, these sites are all part of the axial skeleton, which primarily serves to protect the spinal cord and vital organs. Therefore, the negative prognosis associated with these sites may be related to the involvement of adjacent organs during treatment or the occurrence of complications. This is particularly true for the vertebral region, where lesions can lead to paralysis, significantly impairing the overall condition and quality of life, thus affecting survival rates (11, 51).

In the studies by Liu et al. (45) and Li et al. (51), the laterality of the primary sites did not correlate significantly with PB-DLBCL prognosis. However, the results of the multivariate competing risk analysis in this study show that, regarding tumor laterality, no paired, left, and right are considered protective factors compared to other. On the one hand, it may be because previous studies used traditional COX and Kaplan–Meier analyses, and the different results are due to differences in models. On the other hand, the small number of cases classified as other in this study makes it difficult to rule out potential bias affecting the results (52). Therefore, further validation is needed through studies with larger sample sizes from other databases or multicenter research.

Due to its rarity, there is still no clear consensus on clinical treatment options for PB-DLBCL. Currently, the recommended treatment plan is chemotherapy with rituximab combined with CHOP (53). It is worth noting that surgical treatment in PBL patients with fractures, compression symptoms, or local recurrence can improve prognosis (54). In this study, we also reached the same conclusion that both chemotherapy and systemic treatment demonstrate a protective effect against the incidence of CSD. After controlling for the competing risk event, the risk of CSD in those receiving chemotherapy was reduced by 60% compared to those not receiving chemotherapy, while the risk of CSD in patients receiving systemic treatment was 60.8% compared to those not receiving systemic treatment.

Whether radiotherapy can improve the prognosis of patients with PB-DLBCL remains controversial (11, 55). Multiple previous studies have confirmed that the combination of radiotherapy on the basis of chemotherapy can lead to an improvement in survival prognosis (56–58). It is noteworthy that the aforementioned studies were conducted before the era of rituximab, and they mainly employed anthracycline-based chemotherapy regimens (CHOP schedule) in combination with radiotherapy. However, results from the IELSG-14 study indicated that patients who initially received chemotherapy exhibited significantly better outcomes compared to those who underwent initial radiation therapy, irrespective of whether they subsequently received radiation therapy. Furthermore, the addition of intensive radiation therapy following initial chemotherapy does not enhance treatment outcomes (11). Another study showed that among 1,337 patients diagnosed with PB-DLBCL after 2000, the mean survival was not statistically different between those who received radiotherapy and those who did not receive radiotherapy (59). After 2000, rituximab was widely used in clinical practice, and its excellent targeting of CD20-positive B cells further improved the survival outcomes of patients with PB-DLBCL (60). It is possible that the excellent synergistic effect of rituximab in the treatment of PB-DLBCL has further narrowed the benefits of radiotherapy. Additionally, there has been an increasing concern about the toxicity of radiotherapy, particularly its potential to increase the incidence of second malignancies (61, 62). As a result, in the post-rituximab era, radiotherapy has gradually been overlooked. Similarly, in this study, patients diagnosed between 2000 and 2015 showed that radiation did not demonstrate statistical significance in the multivariable competing risk analysis.

A large number of meta-analyses have confirmed that in the presence of competing risks, utilizing competing risk models yields more accurate results than the Cox and Kaplan-Meier methods (63, 64). The current main chemotherapy regimen for PB-DLBCL is the R-CHOP regimen, in which doxorubicin exhibits significant cardiac toxicity, potentially leading to cardiac dysfunction and an increased risk of developing cardiovascular diseases and atherosclerosis (65). Consequently, the likelihood of CVD among PB-DLBCL patients further escalates, and neglecting the competitive effects would lead to inaccurate results in traditional Cox regression analysis. Due to the aforementioned reasons, this study utilizes CVD as a competing risk event to conduct survival prognosis analysis for patients with PB-DLBCL. Finally, based on the identified independent risk factors, we successfully constructed a competing risk nomogram with excellent predictive ability.

This study has certain limitations. First, although the SEER database provides extensive tumor-related data, some patient information may be missing or incomplete, which could affect the reliability of the analysis results. Second, the data in the SEER database is sourced from specific regions and populations, which may lead to selection bias and consequently affect the external validity of the results. Finally, the SEER database primarily includes demographic information and basic tumor characteristics, lacking in-depth clinical data (such as specific treatment protocols, treatment responses, radiation doses, surgical methods, etc.).

## Conclusion

We utilized publicly available data from the SEER database to successfully construct and validate a nomogram based on a competing risk model, using CSD as the competing risk event, to predict the 1-, 5-, and 10-year CSS for PB-DLBCL patients. This nomogram assists clinicians in making more convenient and accurate prognostic assessments and selecting appropriate treatment strategies.

## Data Availability

Publicly available datasets were analyzed in this study. This data can be found here: https://seer.cancer.gov/.
