# Supplementary figures and images for: Competing risk nomogram for predicting cancer-specific survival in patients with primary bone diffuse large B-cell lymphoma: a SEER-based retrospective study
Source: Front Med (Lausanne). 2025 May 12;12:1572919. doi: 10.3389/fmed.2025.1572919 (PMC12104979; doi:10.3389/fmed.2025.1572919)

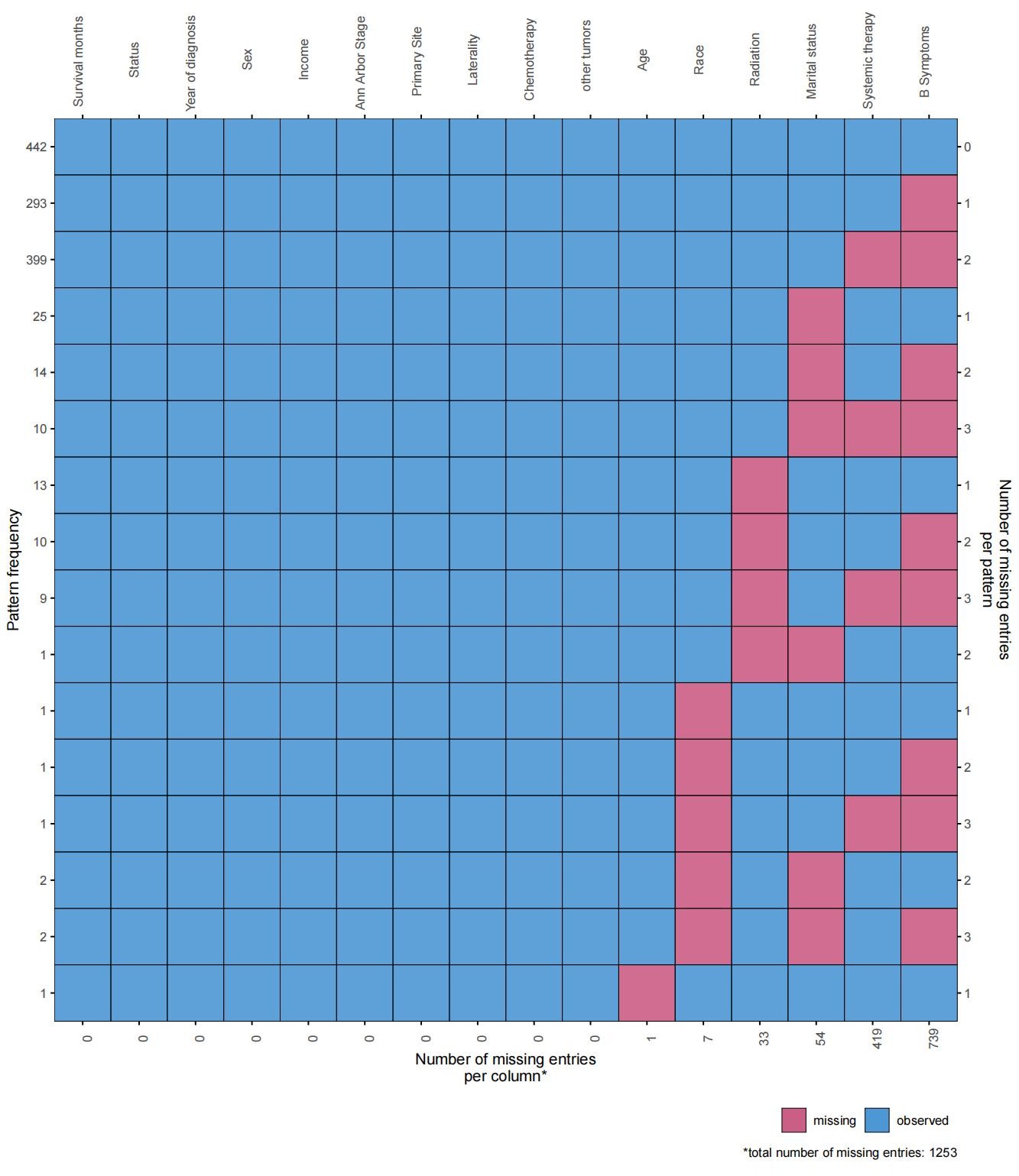


**Figure S1** Detailed Information on missing data

Supplement: Supplementary file 1 [file Supplementary_file_1.docx]
